# A Succinoglycan-Riclin-Zinc-Phthalocyanine-Based Composite Hydrogel with Enhanced Photosensitive and Antibacterial Activity Targeting Biofilms

**DOI:** 10.3390/gels11080672

**Published:** 2025-08-21

**Authors:** Yunxia Yang, Hongmei Zhang, Xueqing Zhang, Shuyan Shen, Baojuan Wu, Dexin Peng, Jie Yin, Yanqing Wang

**Affiliations:** 1 School of Chemistry and Environmental Engineering, Yancheng Teachers University, Yancheng 224007, China; hxzhm@126.com (H.Z.); 19839028643@163.com (X.Z.); ssy1813697159@163.com (S.S.); w03220913@163.com (B.W.); 18862760604@163.com (D.P.); 19551507765@163.com (J.Y.); 2 Jiangsu Province Engineering Research Center of Agricultural Breeding Pollution Control and Resource, Yancheng Teachers University, Yancheng 224007, China

**Keywords:** composite hydrogel, photosensitive property, antibacterial activity, biofilm, metabolomics analysis

## Abstract

Bacterial infections cause serious problems associated with wound treatment and serious complications, leading to serious threats to the global public. Bacterial resistance was mainly attributed to the formation of biofilms and their protective properties. Hydrogels suitable for irregular surfaces with effective antibacterial activity have attracted extensive attention as potential materials. In this study, a succinoglycan-riclin-zinc-phthalocyanine-based composite (RL-Zc) hydrogel was synthesized through an amine reaction within an hour. The hydrogel was characterized via FT-IR, SEM, and rheology analysis, exhibiting an elastic solid gel state stably. The hydrogel showed large inhibition circles on *E. coli* as well as *S. aureus* under near-infrared irradiation (NIR). RL-Zc hydrogel exhibited positively charged surfaces and possessed a superior penetrability toward bacterial biofilm. Furthermore, RL-Zc hydrogel generated abundant single oxygen and mild heat rapidly, resulting in disrupted bacterial biofilm as well as amplified antibacterial effectiveness. A metabolomics analysis confirmed that RL-Zc hydrogel induced a metabolic disorder in bacteria, which resulted from phospholipid metabolism and oxidative stress metabolism related to biofilm disruption. Hence, this study provided a potential phototherapy for biofilm-induced bacterial resistance.

## 1. Introduction

Bacterial infections are the major causes leading to serious complications such as sepsis and diabetes mellitus, as well as various organ failures. Furthermore, antimicrobial resistance coupled with a declining usage of new antibiotics exacerbate this phenomenon invasively. *Enterobacteriaceae*, *Staphylococcus aureus*, *Klebsiella pneumoniae*, *Acinetobacter baumannii*, *Pseudomonas aeruginosa*, and *Escherichia coli* are typical antibiotic-resistant bacteria. The phenomenon in which bacteria escape antibiotic exposure can be divided into two categories: conventionally acquired resistance and intrinsic resistance. Conventionally acquired resistance is characterized by gene mutations mediated by long-term exposures to antibiotics. The horizontal gene transfer by a chromosomal or plasmid is responsible for genetic capabilities and contributes to broader resistance in microbes [1]. The greatest challenge clinically is intrinsic resistance to antibiotics, a complex process that results mainly from biofilm formation for microbial survival. Biofilms provide a strong coating preventing antibiotic invasion and even environmental changes (hypoxia and low pH level) and have contributed to almost 80% of the deaths caused by bacterial infection [2]. Extracellular polymeric substances and quorum sensing contributed to structural integrity and intracellular interaction within bacteria [3]. Microbial biofilm delayed the treatment process significantly associated with chronic wound infection clinically [4]. Thus, the development of effective antimicrobial therapeutics capable of addressing biofilms effectively is imperative.

Due to the complex wound sites as well as wide microbial infections, hydrogels possessing ingenious adaptability have achieved significant progress. Various hydrogels constructed through physical or chemical methods have exhibited substantial antibacterial activity and therapeutic effects via multiple mechanisms [5]. A novel nanocomposite hydrogel was developed based on nanocrystals and nanoparticles, inducing membrane disruption and reactive oxygen species generation [6]. Currently, stimulus-responsive hydrogels have attracted abundant attention for combating bacterial biofilm infections. Temperature, pH, and highly expressed molecules in the microenvironment can control drug release kinetics and ensure safety in physiological conditions [7]. The antibacterial hydrogel was produced for infection disruption and tissue repair coordinately [8]. Lin Li et al. reported a responsive hydrogel targeting endogenous H_2_O_2_, the hydrogel generated high oxidative stress and caused bacteria death as a result [9]. However, the hydrogels required essential factors or antibacterial agents to alter bacterial properties. Moreover, drug release and stability lack precise spatiotemporal monitoring. In contrast, photodynamic therapy (PDT) with precise wavelength control has become more viable. PDT has emerged as a promising non-invasive modality that leads to pathogenic cell destruction and minimizes damage to healthy tissues. The combination of photothermal therapy (PTT) with other therapeutic therapies, such as PDT and antimicrobial peptides, increased its antibacterial efficacy synergically [10,11].

The biological extracellular matrix in hydrogels covered the shortcomings of low solubility, poor adhesion, and significant irritation of photosensitizers. Three-dimensional (3D) structures of hydrogels provided mechanical support and signaling cues of biological behaviors [12]. The hydrogel AC-Gel@Cur-Au was prepared with chemotherapy, PDT, and PTT effects synergistically against bacterial infection [13]. However, the therapeutic efficacy of PDT alone for antibacterial treatment still requires enhancement. The second-generation photosensitizer phthalocyanine (Pc) is particularly notable for high photodynamic efficacy and minimal dark toxicity [14]. The zinc atom acted as the incorporated metal atom of phthalocyanines complexes. Zinc ions formed a diamagnetic closed-shell complexation, which enhanced antibacterial properties and mechanical properties at the same time [14]. Via capitalizing on the remarkable properties of zinc phthalocyanine (Zc) and the unique characteristics of hydrogels, innovative biomedical solutions can be developed.

Non-targeted metabolomics comprehensively provided metabolites in biological samples, thereby revealing potential biomarkers or metabolic pathways. There were only a few studies investigating the metabolic characteristics related to bacterial biofilms. Several metabolic pathways were associated with *P. aeruginosa* biofilm growth, including choline metabolism, alanine metabolism, and TCA cycle [15]. PDT enhanced bacterial inhibition activity and reduced biofilm formation via arginine metabolism [16]. Biofilms are influenced by physiological processes such as PDT and excessive ROS. Microbial metabolites are associated with the restructure of biofilms as a result. Given the significance of PDT therapy and microbial biofilms, it is imperative to thoroughly comprehend the impacts of PDT therapy on their existing metabolites via metabolomics.

**Scheme 1:**
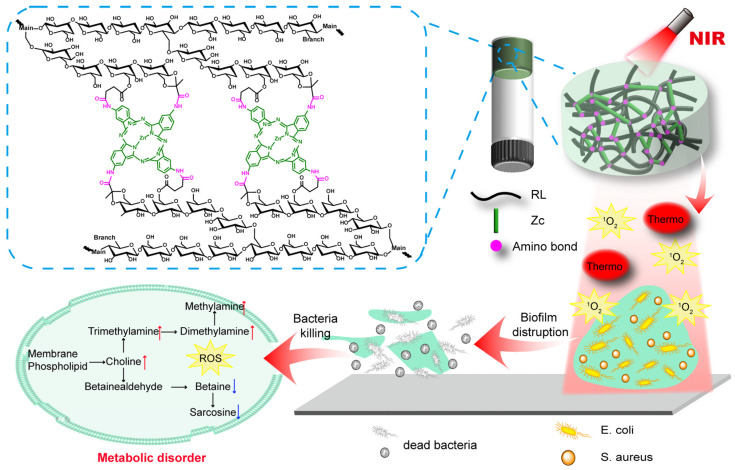
Preparation and mechanism illustration of RL-Zc hydrogel.

Herein, zinc phthalocyanine (Zc) was chemically linked to succinoglycan riclin to obtain hydrogels using a one-step method with potential effects (Scheme 1). We hypothesized that the hydrogel has the potential to inhibit bacterial growth targeting biofilms, inducing metabolic reprogramming as a result. The intrinsic structure and rheological properties of hydrogels were investigated, and antibacterial efficacy was tested against *E. coli* and *S. aureus*. The non-targeted ^1^H NMR-based metabolomics approach was employed to systematically investigate the co-occurrence network between metabolites and biofilms in bacteria. This efficient photosensitive hydrogel will provide valuable insights for the treatment of bacterial infections in future clinical applications.

## 2. Results and Discussion

### 2.1. Synthesis and Characterization of RL-Zc Hydrogels

**Figure 1:**
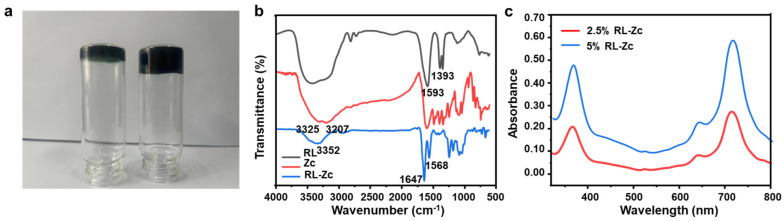
Preparation and characterization of RL-Zc hydrogel. (**a**) RL-Zc hydrogel images; (**b**) FT-IR spectra of RL, Zc, and RL-Zc hydrogel; (**c**) UV–Vis of RL-Zc hydrogels in DMF.

Herein, second-generation photosensitizer Zc was cross-linked with succinoglycan to synthesize hydrogels via a one-pot method. As shown in Figure 1a, RL-Zc hydrogels were obtained with a green color, which was affected by Zc. The hydrogels remained stable when the bottle was upside down, indicating that the hydrogels were successfully synthesized and remained in a gel state. The structure and composition as well as morphology were further characterized in detail. The FT-IR spectra of RL, Zc, and Rl-Zc were shown in Figure 1b. Succinoglycan riclin was composed of glucose and galactose units with pyruvate and succinate substituents. The FT-IR spectrum of RL was identified by asymmetric stretching vibration at 1593 cm^−1^ and symmetric stretching at 1393 cm^−1^ of abundant carboxyl groups in pyruvate and succinate substituents [17,18]. The typical peaks at 3325 and 3207 cm^−1^ were attributed to NH_2_ in photosensitizer Zc [19]. After the synthesis, the presence of new absorption peaks at 1647 cm^−1^ and 1568 cm^−1^ can be assigned to the amide I band and amide II band, respectively [20]. The peak at 3352 cm^−1^ could be attributed to -OH groups of RL in RL-Zc hydrogels [21]. The results confirmed that the amine reaction occurred between the amino groups of Zc and the carboxyl groups of RL. Zc consisted of two characteristic absorption areas at 300–400 nm and 600–800 nm [19]. Consistently, the RL-Zc hydrogels showed strong absorptions at the two regions, and the absorbance intensities were correlated with the hydrogel concentration dissolved in DMF positively (Figure 1c). The results suggested the presence and good stability of Zc in hydrogels.

**Figure 2:**
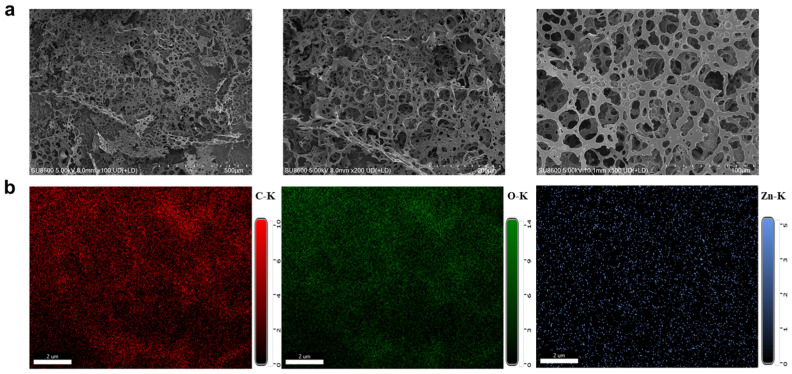
SEM (**a**) and EDS (**b**) images of RL-Zc hydrogels.

Hydrogels have been employed as drug carriers and tissue repair frameworks due to the natural extracellular matrix. The morphology of the freeze-dried RL-Zc hydrogel was analyzed by scanning electron microscopy (SEM) in Figure 2a. The interconnected porous structure was observed, and the different pore sizes resulted in a dense and compact network in hydrogels. The three-dimensional structure of hydrogels provided a dynamic microenvironment for tissue repair and next-generation regenerative medicine [22]. Elemental contents of the hydrogels confirmed the presence of carbon, oxygen, and zinc in hydrogels (Figure 2b), revealing the uniform distribution of Zc and successful synthesis of hydrogels. According to published papers, bacterial biofilms and cell membranes were negatively charged, and positively charged nanoparticles and molecules have been reported to be beneficial for improved penetration and accumulation in biofilms due to electrostatic interaction [23,24]. The abundant amino groups of the zinc phthalocyanine molecules undergo protonation in solution, giving the hydrogels a mean positive charge (16.8 mV) and potential biofilm penetration properties as a result.

### 2.2. Rheology Performance of RL-Zc Hydrogels

**Figure 3:**
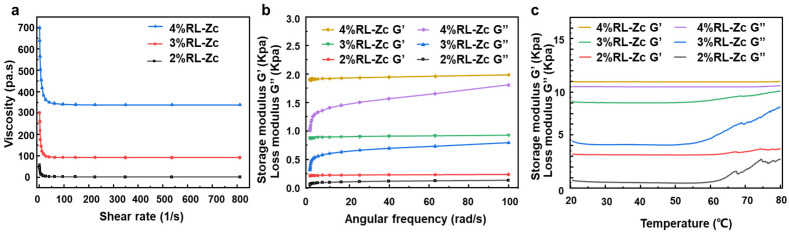
Rheology performance of RL-Zc hydrogels. (**a**) Shear ramp tests; (**b**) dynamic rheological tests; (**c**) oscillatory temperature ramp test.

The shear ramp tests of hydrogels composed of different concentrations of RL were performed, and the curves at different shear rates were illustrated in Figure 3a. As the shear stress increased from 0.1 to 1000 s^−1^, the viscosity of the hydrogels decreased significantly, indicating a shear thinning behavior, which was closely with the injectability of the hydrogel [25]. The viscosity of a polymer is generally related to the mechanical strength [26]. According to the results, the viscosity of hydrogels was positively related to the RL concentration, which may indicate the enhanced strength of hydrogels.

Dynamic rheological measurements were conducted to evaluate the viscoelastic properties of RL-Zc hydrogels. The storage and loss modulus (G′ and G″) versus angular frequency of three prepared hydrogels are shown in Figure 3b; G′ is greater than G″ constantly among all hydrogels, confirming that all hydrogels were at an elastic solid gel state, not a liquid-like flow [25]. Moreover, as the RL concentration increased from 2% to 4%, G’ increased from 0.21 to 1.98 kPa (approximately 9.5-fold), consistent with the increased viscosity mentioned above. Fabricating chemically crosslinked hydrogels has gained attention for improving intermolecular interactions as well as the mechanical properties of hydrogels according to hydrogel design [27]. The increased G’ in the study suggested that the chemical crosslinking in hydrogels improved the mechanical properties of hydrogels significantly, suggesting a much denser crosslinked network formation [28]. The increase in G’’ was observed with elevated frequency in hydrogels, indicating the increased interactions between the polymeric chains in hydrogel frameworks [29].

The oscillatory temperature ramp of hydrogels was tested to investigate the temperature influence on the hydrogels’ viscoelastic properties. Surprisingly, as shown in Figure 3c, storage modulus (G′) was higher than the loss modulus (G″) consistently, and no intersection of the G′ and G″ curves was observed, suggesting that the hydrogels kept a gel state across the whole heating process (20–80 °C). Zc exhibited excellent thermostability, and the amide bond decomposed with a homogeneous rate from 200 °C to 750 °C [29]. Therefore, due to the dense cross-linkages in the hydrogel network, the elastic properties are dominant despite the increase in temperature. The thermal stability of RL-Zc hydrogels suggested their effectiveness in simultaneous PDT and PTT for clinical application under NIR irradiation [30].

### 2.3. Antibacterial Activity of RL-Zc Hydrogels

**Figure 4:**
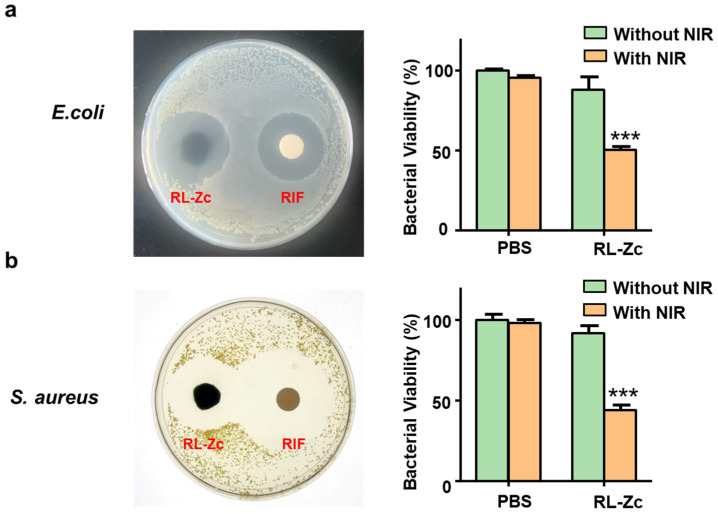
Antibacterial activity of RL-Zc hydrogels. (**a**) Bacterial inhibition circle against *E. coli* under NIR irradiation and positive control RIF (left); bacterial survival viability in co-culture assay (right); (**b**) bacterial inhibition circle against *S. aureus* under NIR irradiation and positive control RIF (left); bacterial survival viability in co-culture assay (right). Data are expressed as mean ± SEM. *** *p* < 0.001 compared with the PBS without NIR group.

Hydrogels have gained extensive attention as medical substitutes for wound dressing in skin repair [31]. The intrinsic antibacterial ability of hydrogels prevented infection and helped the recovery process, promoting wound healing effectively [32]. The antibacterial activity of RL-Zc hydrogels was performed against Gram-negative (*E. coli*) and Gram-positive (*S. aureus*) bacteria through a bacterial inhibition circle and co-culture assays. Succinoglycan riclin and Zc showed low toxicity in the dark according to published papers [33,34]. The hydrogels were spread on the plates, and a known antibiotic rifampicin (RIF) was spread on drug sensitivity papers. RIF exhibited broad-spectrum antibacterial activity, including therapeutic efficacy on most Gram-positive bacteria and limited toxicity on selective Gram-negative species [35]. As illustrated in Figure 4a, bacterial growth in the absence of light was observed on agar plates, indicating that the hydrogels did not show any antimicrobial activity as expected. Zc is the second-generation photosensitizer and generates plentiful singlet oxygen and mild heat rapidly, which originates from NIR [36,37]. Thus, hydrogels showed no antimicrobial capabilities without NIR. Upon NIR irradiation, the hydrogels produced larger diameter inhibition circles for the two target bacteria, displaying superior antimicrobial performance against the two primary pathogenic bacteria related to wound infections. The inhibition circle of RL-Zc hydrogels against *E. coli* was 27.25 ± 0.75 mm, and the inhibition circle against *S. aureus* was 26.25 ± 0.85 mm (Table S1). The sizes of the inhibition circles were similar between the two species. The lipopolysaccharide (LPS) layer of *E. coli* limited the transport of RIF across the membrane [38]. Therefore, RIF showed significant differences against two species in Table S1, consistent with the results as reported [35,38]. However, low solubility, instability, and antibiotic resistance limited its clinical usage [35]. RL-Zc hydrogels exhibited superior antibacterial on both strains and acted as potential drug-loading materials for combination therapies.

The antibacterial activity of hydrogels was further evaluated by a co-culture assay in vitro. After a 12 h co-incubation period of bacteria with or without hydrogels/NIR irradiation, the bacterial survival results were summarized in Figure 4b. Individual NIR irradiation without RL-Zc hydrogels exhibited no antibacterial effect, consistent with the evidence of no significant effect on the photothermal performance in PBS [39]. Consistently, the viability of the two bacteria showed no significant alteration in the dark. Treatment of hydrogels with NIR irradiation for 10 min caused a 50% reduction in CFU, confirming the strong anti-bacterial effects of RL-Zc hydrogels under NIR conditions.

### 2.4. Bacterial Disruption and Biofilm Penetration of RL-Zc Hydrogels

**Figure 5:**
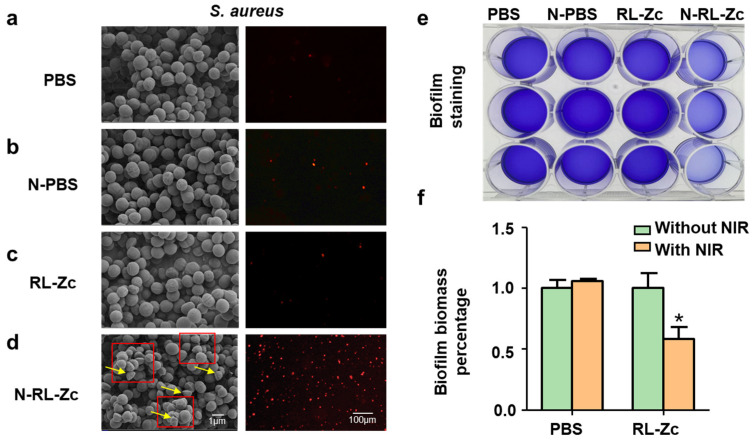
Bacterial disruption and biofilm penetration of RL-Zc hydrogels against *S. aureus*. (**a**–**d**) Representative SEM images and PI staining images of *S. aureus*; yellow arrows showed membrane wrinkling and structural damage, red boxes showed extensive bacterial death; (**e**,**f**) representative images of the biofilms against *S. aureus* stained by crystal violet in three independent triplicates and quantification of biofilm biomass. Data are expressed as mean ± SEM. * *p* < 0.05, compared with the PBS without NIR group.

To investigate the morphology and membrane integrity, *S. aureus* was examined using SEM in Figure 5a. The bacteria in the three groups were spherical with intact morphology; the cells gathered together and distributed evenly (Figure 5a–c), while hydrogels with NIR irradiation induced membrane wrinkling and structural damage (marked in yellow arrow), resulting in extensive bacterial death (marked in red boxes) (Figure 5d). Moreover, propidium iodide (PI) staining experiments were also added to investigate bacterial membrane damage and cell death [40]. The fluorescent dye propidium iodide (PI) stains only cells with damaged cell membranes, thereby enabling the selective labeling of dead cells. As shown in the right column in Figure 5a–e, the bacteria in the first three groups exhibited negligible red fluorescence, while the samples under RL-Zc hydrogels with NIR exhibited extensive red fluorescence, demonstrating the compromised integrity of bacterial cell membranes.

The structural complexity of microbial biofilm provided a matrix for the pathogenic bacteria to evade the immune system of the host [41]. To demonstrate the complex structure of the biofilm associated with antibacterial mechanisms, biofilm formation was verified using the crystalline violet staining. Xiao-Ling Lei et al. reported that a pH-responsive micelle eliminated bacterial biofilms and accelerated antibacterial performance [42]. As shown in Figure 5e, the bacteria exhibited mature biofilm accumulation within 48 h, bacterial biofilm formation was intact upon NIR irradiation alone. A slight reduction in biofilm qualification was observed in RL-Zc hydrogels without light, which might be related to the presence of hydrogels, which occupied the biofilm position as a result. Significantly, RL-Zc hydrogels under NIR irradiation inhibited almost 50% of biofilm formation and subsequently caused membrane contraction as well as bacteria disruption (Figure 5f). In addition, similar results were demonstrated in Gram-negative bacteria *E. coli* in Figure S3. The fluorescent dye propidium iodide (PI) was visualized clearly after RL-Zc hydrogel treatment with NIR irradiation, suggesting that bacteria were damaged with cell penetration. Biofilm biomass was significantly reduced by 50%, consistent with the cell survival rate mentioned above. The results indicated that biofilm penetration was the major mechanism contributing to the antibacterial activity of RL-Zc hydrogels with PDT.

### 2.5. Photosensitive Property of RL-Zc Hydrogels

**Figure 6:**
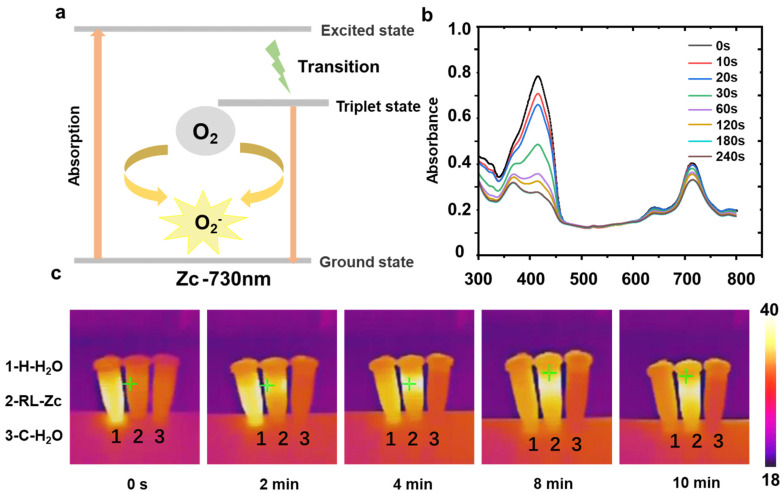
Photosensitive property of RL-Zc hydrogels. (**a**) Activation pathways of the photosensitizers Zc; (**b**) UV–Vis of singlet oxygen production of RL-Zc hydrogels under NIR irradiation; (**c**) the infrared thermal images of RL-Zc hydrogels under NIR irradiation. The green cross indicated the center point of the image.

NIR possessed the strongest ability for light penetration through tissue, making it suitable for medical applications [30]. The release of Zc promoted PDT for cancer therapy with excellent singlet oxygen quantum yield [43]. As shown in Figure 6a, Zc absorbed photons of NIR, causing electrons to transition from the ground state to the excited state, which underwent a radiative transition to a longer-lived triplet excited state within the microsecond range. Subsequently, Zc in the triplet excited state (T_1_) transferred energy to surrounding triplet oxygen molecules (^3^O_2_), transforming them into highly reactive singlet oxygen (^1^O_2_). To further validate the potential of the RL-Zc hydrogel as a PDT agent, the production of singlet oxygen under NIR was determined using DPBF as a chemical quencher. As expected, RL-Zc hydrogels possessed the superior photodegradation of DPBF within 240 s under laser irradiation (Figure 6b), indicating the rapid production efficiency of ^1^O_2_ across the whole study. The generation efficacy was associated with the Zc concentration as well as NIR intensity [30]. The hydrogels in dark conditions exhibited an inability to generate singlet oxygen (Figure S1). Thus, Zc represented a typical group of photosensitizers applicable to generate single oxygen effectively, disrupting biomolecules and triggering cell death [36,37].

A photothermal analysis of the RL-Zc hydrogel was performed under a 730 nm laser at room temperature (25 °C). As illustrated in the infrared thermal images (Figure 6b), the left sample was hot water at 40 °C, for high temperature indication in the initial period of the study. The temperature of the hot water decreased significantly at room temperature. The RL-Zc hydrogel solution (2.5% wt) rapidly increased from 25 °C to 39 °C upon NIR irradiation over 10 min. The heat below 42 °C enabled effective low-temperature photothermal therapy [10]. The mild heat in low-temperature PTT damaged their essential biomolecules and structures, thereby offering a solution to penetrate biofilms and induce bacterial death [43]. In contrast, the temperature of the control solution only increased slightly. Consequently, RL-Zc hydrogels were not only effective in photodynamic therapy, but also served as photothermal agents, resulting in damaged biofilms and enhanced antibacterial activity.

Briefly, compared with traditional antimicrobials, positively charged RL-Zc hydrogels could easily be associated with bacterial membranes via electrostatic adsorption. Moreover, zinc phthalocyanine molecules generated plentiful singlet oxygen and mild heat rapidly, which originated from near-infrared light energy [36,37]. The multi-target nature of excess ^1^O_2_ induced peroxidized biofilms and reduced biofilm biomass [44,45]. Photothermally heating embedded with phthalocyanine dyes efficiently perturbed the lipid bilayers in the cell membrane, resulting in increased cell permeability [46]. Singlet oxygen is capable of crossing the cell membrane barrier and can degrade both the bacterial cell membrane as well as nucleic acid [47,48]. Combining PDT and PTT effects, RL-Zc hydrogels disrupted bacterial biofilm as well as amplified antibacterial effectiveness.

### 2.6. Metabolic Profiles of S. aureus Based on ^1^H NMR Technique

To further associate the effects of hydrogels with bacterial metabolism, metabolomics analysis was employed on *S. aureus* treated with NIR and NIR-treated hydrogels, followed by a combined multivariate analysis. Representative ^1^H NMR spectra with total metabolites signals of *S. aureus* were shown in Figure S2a; a total of 24 metabolites were identified and assigned according to databases and related papers. The metabolites between the PBS and NIR+PBS groups displayed high similarity, including amino acids, organic acids, sugars, and amine derivatives. The metabolites in the NIR+RL-Zc groups exhibited differences from the other two groups. Consistently, the PCA score plot shown in Figure S2b confirmed the metabolic alterations among the three groups. The samples in the PBS group and NIR+PBS group were overlapped, indicating that absolute NIR in a short time did not impact the metabolites significantly. The *S. aureus* preserved in NIR+RL-Zc groups were clustered but clearly separated from the two groups, suggesting that the metabolic profiles were severely perturbed under NIR and the hydrogels.

**Figure 7:**
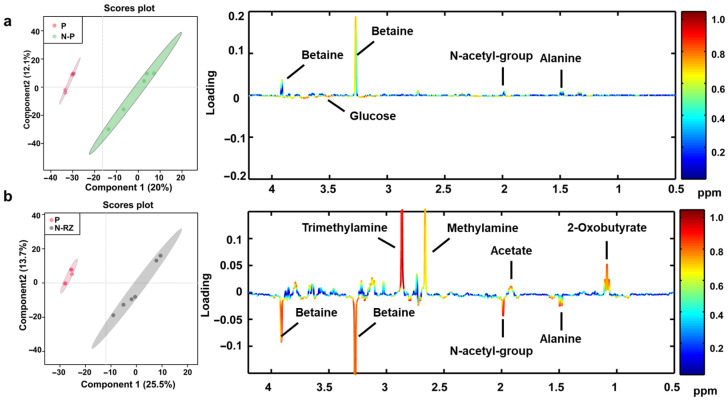
PLS-DA analysis of metabolomics profiles. (**a**) Score plots and color-coded loading plots of 1H NMR spectra between the PBS group and the N-PBS group. (**b**) Score plots and color-coded loading plots of 1H NMR spectra between PBS group and N-RZ group.

To further demonstrate the metabolites alterations in detail, the assigned metabolites with characteristic peaks were analyzed in Figure 7. No obvious alterations were observed between PBS and NIR+PBS groups (Figure 7a). However, the NIR and RL-Zc hydrogels treated at the same time induced severe metabolic changes in the bacteria; the metabolites were color-coded according to the model correlation coefficient in Figure 7b. The detained metabolic alterations were quantified in Table 1 shown below. Overall, the concentration of metabolites presented in *S. aureus* included most amino acids, organic acids, sugars, and amine derivatives. Only four metabolites (alanine, glucose, betaine, and sarcosine) displayed differences from the *S. aureus* in the PBS group. Marked increased levels of 2-oxobutyrate, acetate, methylamine, dimethylamine, trimethylamine, choline, glucose, and myo-inositol and decreased levels of isoleucine, valine, leucine, alanine, glutamine, and betaine were observed in the NIR+RL-Zc group. The metabolic pathways disturbed here were closely associated with three metabolic pathways: phospholipid metabolism, oxidative stress metabolism, and amino acid metabolism.

Metabolomics provides insights into an organism’s physiological state and the impact of external agents on metabolic pathways. Disruption of biofilms provided opportunities for the cell penetration of excess ^1^O_2_ and mild heat. Photothermally heating embedded with phthalocyanine dyes efficiently perturbed the lipid bilayers in the cell membrane, resulting in increased cell permeability [46]. As major components in cell membranes as well as biofilms, phospholipids metabolism would be disturbed, accompanied by deterioration of structural membranes [49]. The numerous compounds related to the phospholipids metabolism were disturbed with the disruption of membrane integrity, including choline and trimethylamine (TMA) metabolism [50]. TMA was further oxidized to dimethylamine and methylamine as a result [51]. In the study, high elevations of choline and downstream TMA metabolites were observed in the *S. aureus* samples after NIR and RL-Zc treatments, confirming that the biofilms and the cell membranes were disrupted, contributing to cell damage. Furthermore, choline-based ionic liquids with amino acids exhibited antibiofilm activity and antibacterial activity [52]. Thus, choline and TMA metabolism may be potential biomarkers for biofilm disruption and bacterial death.

The photosensitive assay suggested that hydrogels induced abundant ^1^O_2_ in bacteria, which caused the bacteria to dissipation. Betaine was the most abundant metabolite in the study and acted as an effective antioxidant in vivo [53]. The significantly decreased level of betaine in the NIR+RL-Zc group could be attributed to its excessive consumption to counteract the abundant ^1^O_2_. Moreover, decreased betaine was related to the damaged membrane and enhanced choline metabolites mentioned above. Accumulation of oxidative stress and heat disrupted membrane homeostasis and promoted bacterial death as a result. As another betaine derivative, sarcosine was also reduced to alleviate the severe oxidative stress in the NIR+RL-Zc group [54]. Obvious alterations among amino acids were demonstrated in the bacteria under NIR+RL-Zc stress. Branched-chain amino acids (BCAAs) and glutamine are essential amino acids in vivo. The NIR+RL-Zc treatments introduced bacterial dysfunction, which was evidenced by the significant disturbance of amino acid metabolism. The metabolic disorder revealed the cell damage of RL-Zc hydrogels in turn, and the approach is increasingly employed to identify antimicrobial targets [55]. Additionally, ^1^H NMR-based metabolomics provided core metabolites for constructing metabolic maps, while the method may miss low-abundance metabolites. Integrating other metabolomics methods and clarifying more metabolites could indeed complement the findings and enhance the safety profile in further applications.

## 3. Conclusions

In summary, a potential RL-Zc hydrogel capable of employing a photosensitive strategy has been developed to treat bacterial infections. The RL-Zc hydrogel was synthesized via an amide reaction between succinoglycan riclin and zinc phthalocyanine rapidly. RL-Zc hydrogel possessed a positive charge and inhibited bacterial activity under laser irradiation. The RL-Zc hydrogel effectively produced single oxygen and mild heat, offering a promising approach for bacterial biofilm as well as membrane disruption. RL-Zc hydrogel led to a metabolic disorder, consequently exacerbating bacterial death. The RL-Zc hydrogels in the antibacterial practices in this study represent a significant step forward. Further in vivo efficacy and safety studies, broader spectrum testing, and mechanistic studies are crucial for future applications. RL-Zc hydrogel showed no activity in the absence of NIR laser irradiation, indicating that the hydrogel was suitable as a drug carrier. The optimization of hydrogel in drug loading and release needs further exploration. This study has provided a novel photosensitive hydrogel targeting bacterial biofilm, illustrating its potential application for infected wound healing.

## 4. Materials and Methods

### 4.1. Materials

Zinc phthalocyanine (Zc) was purchased from Yanshen Technology Co., Ltd. (Changchun, China). Succinoglycan riclin was produced from Agrobacterium sp. ZCC3656, after which followed a purification by the Sevag method [56]. The purity of succinoglycan was above 95% in this study. *Staphylococcus aureus* (*S. aureus*, 1.6750) and *Escherichia coli* (*E. coli*, 1.12882) were purchased from China General Microbiological Culture Collection Center (Beijing, China). The regents used here, including 1,3-diphenylisobenzofuran (DPBF), crystal violet, 1-(3-Dimethylaminopropyl)-3-ethylcarbodiimide (EDC), N-Hydroxysuccinimide (NHS), N,N-Dimethylformamide (DMF), and rifampicin (RIF) were purchased from Aladdin Chemistry Co., Ltd. (Shanghai, China). MES butter was purchased from Yuanye Bio-Technology Co., Ltd. (Shanghai, China).

### 4.2. Preparation of Succinoglycan Riclin-Zinc Phthalocyanine (RL-Zc) Hydrogels

The hydrogels were prepared via a covalently cross-linked method, and the hydrogels can be produced within an hour. Briefly, succinoglycan riclin was dissolved in a 0.05 M MES buffer (pH 5.5) to result in a 2% solution. The carboxyl group in succinoglycan riclin was activated via a EDC/NHS coupling method for 20 min at room temperature [57]. EDC and NHS were added to the solution with a molar ratio of 2:5:1 in order, with a 15 min interval according to product instructions (Thermo Scientific, Waltham, MA, USA) [58]. Sodium bicarbonate (final concentration was 0.5M when dissolved in water) was dropped into the solution slowly to adjust pH to 7.0. Crosslinking was conducted via adding Zc at a molar ratio, with succinoglycan riclin, of 1:4 and stirring for 20 min. RL-Zc hydrogel networks were produced for 5 min and followed by immersion in deionized water three times to remove any excess reagents.

### 4.3. Fourier Transform Infrared Spectroscopy (FT-IR)

Hydrogels were frozen and moved to the freeze dryer (Songyuan Huaxing Technology, Beijing, China). The dried hydrogels were analyzed via a Vertex80/RamanII FT-IR spectrometer (Bruker, Karlsruhe, Germany). Fourier-transformed infrared analysis provided chemical characterization of fabricated cross-linking hydrogels. The wavenumber was recorded at a range of 500–4000 cm^−1^.

### 4.4. UV–Vis Spectroscopy

The prepared hydrogels were dispersed into DMSO/DMF for 24 h, accompanied by ultrasonic technology [19]. The UV–Vis spectra were obtained in the wavelength range of 400–800 nm using UV–Vis spectroscopy (Perkin-Elmer, Norwalk, CT, USA).

### 4.5. Scanning Electron Microscopy (SEM) Analysis

A Quattro environmental scanning electron microscope (SU8600, Hitachi, Tokyo, Japan) was employed to investigate the morphological properties of the freeze-dried hydrogels. Energy Dispersive X-ray Spectroscopy (EDS) (Hitachi, Tokyo, Japan) was applied to determine the elemental structure in the samples.

### 4.6. Zeta Potential Measurement

The surface charge of the RL-Zc hydrogel was assessed by the Zeta potential. The prepared hydrogel was dispersed into H_2_O for 24 h, accompanied by magnetic stirring. The zeta potential was measured via Litesizer LS500 (Anton Paar GmbH, Graz, Austria).

### 4.7. Rheology Analysis

The succinoglycan riclin concentration was altered by 2%, 3%, and 4% for different hydrogels. The viscosity and viscoelastic performance of the hydrogels were investigated on a rheometer (DHR-1, Waters, Milford, Massachusetts, USA) [20]. All the experiments were performed using a parallel plate with a plate gap of 1000 μm. The viscosity of the hydrogels contained different concentrations of succinoglycan riclin under a controlled shear rate from 0.1 s^−1^ to 1000 s^−1^. Hydrogels or viscose solutions varied the storage modulus (G′) and loss modulus (G″) numbers accordingly. The plots of G′ and G″ versus angular frequency were determined with a constant strain. Moreover, temperature ramp tests were considered from 20 to 80 °C with a constant heating rate.

### 4.8. Antibacterial Assay

#### 4.8.1. Bacterial Culture

*E. coli* and *S. aureus* were cultured in Luria–Bertani (LB) and Tryptic Soy Broth (TSB) media at 37 °C, respectively. Live bacteria during the logarithmic growth phase were gathered and randomly divided into 12-well plates (10^8^ CFU/well). The antibacterial effects were conducted in four experimental groups: control group (PBS), PBS+NIR group, Rl-Zc group, and Rl-Zc group+NIR laser group. The hydrogels were planked in wells in advance and treated with or without NIR irradiation (730 nm, 1 W/cm^2^) for 10 min. After a 12 h incubation at 37 °C, the OD600 was determined for bacterial growth. The antibacterial activity was also determined via the spread plate assay. About 100 μL of bacterial suspension (10^6^ CFU/mL) was gradient diluted on agar plates, and hydrogels were spread on the plates, followed by NIR irradiation for 10 min and incubation for 12 h overnight. RIF solution (10 μg/mL) was spread on drug sensitivity papers (10 mm) before being placed on agar plates.

#### 4.8.2. Bacterial Morphology

*S. aureus* in 12-well plates after various treatments above were gathered in tubes and washed for 2 times with 0.1 M PBS. A pre-cold 2.5% glutaraldehyde solution was added along the tube wall slowly and fixed the bacteria overnight. The samples were rinsed with 0.1M PBS three times and dehydrated with gradient concentrations of ethanol solution (including 30%, 50%, 70%, 80%, 90%, and 100%) for 15 min each time. The samples were then treated with a mixture of ethanol and isoamyl acetate (*v*/*v* = 1/1) for 30 min and pure isoamyl acetate for 1 h in order. The samples were dried at the critical point and coated before observation via scanning electron microscope.

#### 4.8.3. Bacterial Biofilm Assessment

The bacterial biofilm was assessed via crystal violet assay. The bacteria in logarithmic growth phase were incubated in 12-well plates (10^8^/well) without shaking for 48 h for bacterial formation. The upper medium was replaced, and biofilms were washed with PBS three times to remove planktonic bacteria. The biofilms were incubated with distributed hydrogels for 12 h without shaking. The upper suspension was removed and washed with PBS; the biofilms were fixed with 95% ethanol for 15 min. After staining with 500 μL of 0.5% crystal violet solution for 5 min, 33% acetic acid was added before the absorbance measurement at 590 nm according to a published paper [9].

#### 4.8.4. PI Staining

PI staining was applied to investigate the effects of hydrogels on cell membrane damage and cell death. Live bacteria were treated with or without RL-Zc hydrogels and NIR irradiation (730 nm, 1 W/cm^2^) for 10 min. After 12 h incubation at 37 °C, the bacteria were gathered and washed with PBS. The samples were then incubated with PI in the dark for 10 min. Finally, the stained bacterial cells were observed using a fluorescence microscope (NIB 600, Jiangnan Novel Optics, Nanjing, China).

### 4.9. Singlet Oxygen Generation

Singlet oxygen generation was quantified via 1,3-diphenylisobenzofuran (DPBF, Aladdin, Shanghai, China) as a chemical quencher. The reduction in the DPBF concentration with the irradiation time was determined at 417 nm with NIR irradiation (730 nm). DPBF has a characteristic absorption peak at a specific wavelength, which weakens when singlet oxygen reacts with it. Therefore, the respective DPBF absorbance values against irradiation time give a straight line whose slope indicates the photosensitizer potency to produce singlet oxygen.

### 4.10. Photothermal Performance

The prepared hydrogels were dispersed into H_2_O for 24h and placed in 1 mL transparent, plastic centrifugal tubes. Hot water and cold water were used as high-temperature indication and control samples, respectively. These samples were then irradiated using a 730 nm laser with an intensity of 1 W/cm^2^. A thermal imaging camera (Mettler Toledo, Zurich, Switzerland) was employed to take the thermal changes in the samples.

### 4.11. ^1^H NMR-Based Metabolomics

*S. aureus* was cultivated in a 6-well plate and treated with PBS or hydrogels with NIR irradiation as described above. The ^1^H NMR-based metabolomics was performed according to papers published [18]. Briefly, bacteria were harvested by centrifugation at 3000 rpm and then washed with precooled PBS. The metabolites were extracted with methanol/H_2_O (3.8 mL, v: *v*/*v* = 1:0.9) and homogenized via sonication for 4 min intermittently. The chloroform was added to the samples and extracted for 10 min at 4 °C. The organic solvents of the upper layer were removed under nitrogen. The remaining samples were lyophilized and resolved in D_2_O with TMSP as a reference. A ^1^H NMR spectrometer (Bruker AVANCE III, 400 MHz) (Bruker, Karlsruhe, Germany) was used at 298 K. All ^1^H NMR spectra were analyzed via multivariable statistics, including principal component analysis (PCA) and partial least squares discriminant analysis (PLS-DA). The metabolites were identified according to various data banks. The metabolites were qualified, and fold changes were presented according to the normalized bins.

### 4.12. Statistical Analysis

All data were expressed as means ± SEM. Data were analyzed using GraphPad Prism 9, and data among different groups were compared by one-way ANOVA with Tukey post-hoc tests. Differences with *p* < 0.05 were considered statistically significant.

## Figures and Tables

**Table 1:** Potential marker metabolites identified by ^1^HNMR and their variations among groups.

No.	Metabolite	Assignments	N-P/P		N-RL/P	
			FC ^1^	*p*-Value ^2^	FC	*p*-Value
1	Isoleucine	δ-CH3, γ-CH3, α-CH	1.33		0.32	**
2	Leucine	δ-CH3, δ-CH3, α-CH	1.24		0.58	*
3	Valine	γ-CH3, γ-CH3, α-CH	1.24		0.21	***
4	2-Oxobutyrate	CH3, CH2	1.4		14.8	**
5	Lactate	CH3	1.32		1.01	
6	Alanine	CH3, CH	1.37	*	0.52	***
7	Lysine	δ-CH2, β-CH2, ε-CH2, α-CH	0.95		1.15	
8	Acetate	CH3	1.03		3.45	**
9	N-Acetyl group	CH3, CH2	1.37	*	0.38	***
10	Glutamate	β-CH2, γ-CH2, α-CH	1.05		1.12	
11	Glutamine	βCH_2_, γCH_2_, αCH	0.99		0.46	**
12	Succinate	CH2	1.18		0.86	
13	Methylamine	CH3	0.71		18.11	*
14	Sarcosine	CH3	1.35	*	0.59	**
15	Dimethylamine	CH3	1.3		3.71	**
16	Trimethylamine	CH3	1.12		17.73	***
17	Creatine	CH3, CH2	1.21		1.89	*
18	Choline	CH3	0.6		3.01	**
19	Betaine	CH2, CH3	1.37	*	0.65	**
20	Myo-inositol	CH3	1.15		1.42	*
21	Glycine	CH2	1.13		1.53	
22	Glucose	CH, CH, CH	0.85	**	1.17	*
23	Phenylalanine	CH=CH	1.23		1.3	
24	Formate	CH	1.06		1.72	

^1^ Color coded according to fold change value, red indicated the increased and blue rep-resented the decreased concentrations of metabolic components in compared groups. Color bar 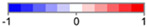. ^2^ *p*-Values were calculated based on a parametric Student’s *t*-test (dependent on the conformity to the normal distribution). * *p* < 0.05, ** *p* < 0.01, *** *p* < 0.001.

## Data Availability

The original contributions presented in this study are included in the article. Further inquiries can be directed to the corresponding authors.

## Supplementary Materials

The following supporting information can be downloaded at: https://www.mdpi.com/article/10.3390/gels11080672/s1, Figure S1: UV–Vis of singlet oxygen production of RL-Zc hydrogels without NIR irradiation. Figure S2: (a) Representative ^1^H NMR spectra with total metabolite signals of *S. aureus* and (b) PCA score plot among the three groups. Figure S3: Bacterial disruption and biofilm penetration of RL-Zc hydrogels against *E. coli*.: (a–d) Representative PI staining images of *E. coli* and (e,f) representative images of the biofilms against *E. coli* stained by crystal violet in three independent triplicates and quantification of biofilm biomass. Table S1: Inhibition circle sizes of RL-Zc hydrogels and positive control RIF against *E. coli* and *S. aureus.*

## Abbreviations

The following abbreviations are used in this manuscript:

RL	Riclin
Zc	Zinc phthalocyanine
PDT	Photodynamic therapy
PTT	Photothermal therapy
Pc	Phthalocyanine
PCA	Principal component analysis
PLS-DA	Partial least squares discriminant analysis
SEM	Scanning electron microscopy
FT-IR	Fourier transform infrared spectroscopy
NIR	Near-infrared irradiation
PI	Propidium iodide
RIF	Rifampicin
