# Supplementary material for: A Succinoglycan-Riclin-Zinc-Phthalocyanine-Based Composite Hydrogel with Enhanced Photosensitive and Antibacterial Activity Targeting Biofilms
Source: Gels. 2025 Aug 21;11(8):672. doi: 10.3390/gels11080672 (PMC12385968; doi:10.3390/gels11080672)
Supplement: Supplementary file 1 [file gels-11-00672-s001.zip › gels-3791172-supplementary.pdf]

A Succinoglycan-Riclin-Zinc-Phthalocyanine-Based Composite Hydrogel with Enhanced Photosensitive and Antibacterial Activity Targeting Biofilms

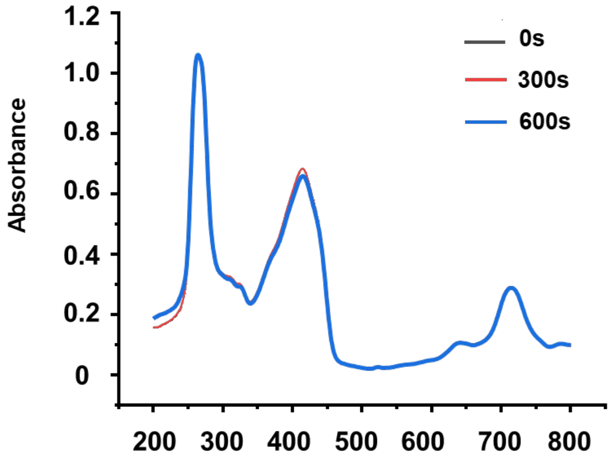

Figure.S1. Uv-vis of singlet oxygen production of RL-Zc hydrogels withour NIR irradiation.

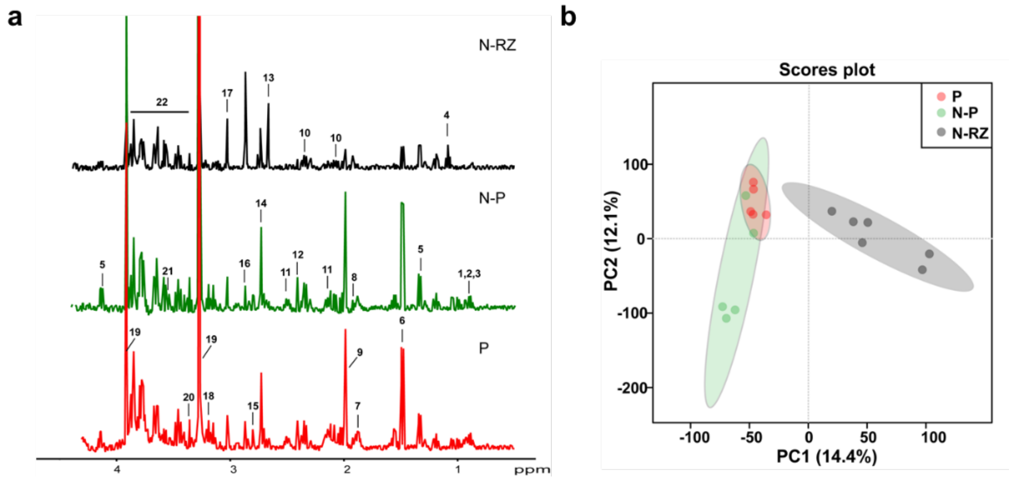

Figure S2. (a) Representative ^{1}H NMR spectra with total metabolites signals of *S. aureus*. (b) PCA score plot among the three groups.

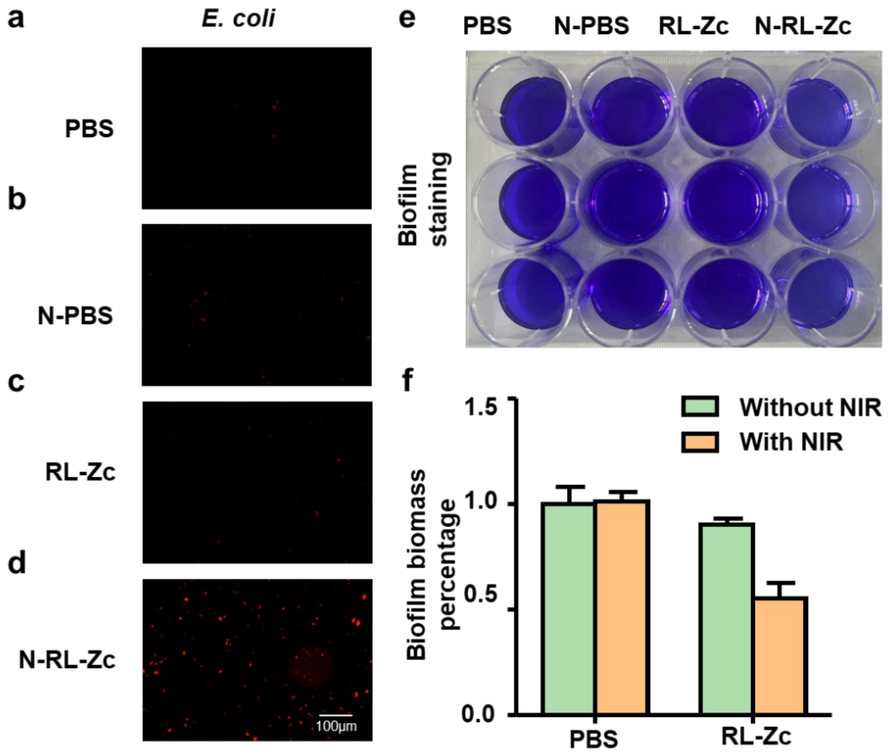

Figure S3. Bacterial disruption and biofilm penetration of RL-Zc hydrogels against *E. coli*. (a-d) Representative PI staining images of *E. coli*; (e-f) Representative images of the biofilms against *E. coli* stained by crystal violet in three independent triplicates and quantification of biofilm biomass.

Table S1. Inhibition circle sizes of RL-Zc hydrogels and positive control RIF against *E. coli* and *S. aureus*.

	RL-Zc	RIF (10 μg/mL)
E. coli	27.25±0.75	21.50±1.04
S. aureus	26.25±0.85	40.50±1.56
